# PD-L1 Expression after Neoadjuvant Chemotherapy in Triple-Negative Breast Cancers Is Associated with Aggressive Residual Disease, Suggesting a Potential for Immunotherapy

**DOI:** 10.3390/cancers13040746

**Published:** 2021-02-11

**Authors:** Beatriz Grandal, Manon Mangiardi-Veltin, Enora Laas, Marick Laé, Didier Meseure, Guillaume Bataillon, Elsy El-Alam, Lauren Darrigues, Elise Dumas, Eric Daoud, Anne Vincent-Salomon, Laure-Sophie Talagrand, Jean-Yves Pierga, Fabien Reyal, Anne-Sophie Hamy

**Affiliations:** 1 Residual Tumor & Response to Treatment Laboratory, RT2Lab, Translational Research Department, INSERM, U932 Immunity and Cancer, University Paris, 75005 Paris, France; beatriz.grandalrejo@curie.fr (B.G.); enora.laas@curie.fr (E.L.); elise.dumas@curie.fr (E.D.); eric.daoud@curie.fr (E.D.); anne-sophie.hamy-petit@curie.fr (A.-S.H.); 2 Department of Surgical Oncology, Institut Curie, University Paris, 75005 Paris, France; manon.mangiardi@curie.fr (M.M.-V.); lauren.darrigues@curie.fr (L.D.); laure.talagrand@curie.fr (L.-S.T.); 3 Department of Pathology, Henri Becquerel Cancer Center, INSERM U1245, UniRouen Normandy University, 76038 Rouen, France; marick.lae@chb.unicancer.fr; 4 Department of Pathology, Institut Curie, University Paris, 75005 Paris, France; didier.meseure@curie.fr (D.M.); guillaume.bataillon@curie.fr (G.B.); elsy.elalam@curie.fr (E.E.-A.); anne.salomon@curie.fr (A.V.-S.); 5 Department of Medical Oncology, Institut Curie, University Paris, 75005 Paris, France; jean-yves.pierga@curie.fr

**Keywords:** breast cancer, neoadjuvant chemotherapy, PD-L1, immunohistochemistry, residual cancer burden, residual disease, immunotherapy, immune checkpoint inhibitor

## Abstract

**Simple Summary:**

Immune checkpoint inhibitors (ICI) are now part of the therapeutical arsenal for cancers at several sites and in several settings. PD-L1 expression is assessed to predict treatment response. We used immunohistochemistry (E1L3N clone) to assess PD-L1 expression on tumor and immune cells from a cohort of 89 surgical specimens of T1-T3NxM0 triple-negative breast cancers (TNBC) from patients treated with neoadjuvant chemotherapy (NAC) with residual disease. PD-L1 expression levels were low in tumor and immune cells from post-NAC surgical specimens. PD-L1 positivity in tumor cells was significantly associated with aggressive post-NAC tumor characteristics. A small subset of TNBC patients displaying PD-L1 expression in the context of a more extensive post-NAC tumor burden could benefit from ICI treatment after NAC.

**Abstract:**

The consequences of neoadjuvant chemotherapy (NAC) for PD-L1 activity in triple-negative breast cancers (TNBC) are not well-understood. This is an important issue as PD-LI might act as a biomarker for immune checkpoint inhibitors’ (ICI) efficacy, at a time where ICI are undergoing rapid development and could be beneficial in patients who do not achieve a pathological complete response. We used immunohistochemistry to assess PD-L1 expression in surgical specimens (E1L3N clone, cutoff for positivity: ≥1%) on both tumor (PD-L1-TC) and immune cells (PD-L1-IC) from a cohort of T1-T3NxM0 TNBCs treated with NAC. PD-L1-TC was detected in 17 cases (19.1%) and PD-L1-IC in 14 cases (15.7%). None of the baseline characteristics of the tumor or the patient were associated with PD-L1 positivity, except for pre-NAC stromal TIL levels, which were higher in post-NAC PD-L1-TC-positive than in negative tumors. PD-L1-TC were significantly associated with a higher residual cancer burden (*p* = 0.035) and aggressive post-NAC tumor characteristics, whereas PD-L1-IC were not. PD-L1 expression was not associated with relapse-free survival (RFS) (PD-L1-TC, *p* = 0.25, and PD-L1-IC, *p* = 0.95) or overall survival (OS) (PD-L1-TC, *p* = 0.48, and PD-L1-IC, *p* = 0.58), but high Ki67 levels after NAC were strongly associated with a poor prognosis (RFS, *p* = 0.0014, and OS, *p* = 0.001). A small subset of TNBC patients displaying PD-L1 expression in the context of an extensive post-NAC tumor burden could benefit from ICI treatment after standard NAC.

## 1. Introduction

Breast cancer (BC) remains the most frequent and deadly cancer in women [1]. Triple-negative (TNBC) subtypes account for 10% to 20% of all BCs, are more aggressive than other subtypes and are associated with a poorer prognosis [2]. Historically, chemotherapy was the only viable systemic treatment option for both local and advanced TNBC. However, the arrival of poly-ADP ribose polymerase (PARP) inhibitors, antiandrogen therapy and immunotherapy is gradually opening up new prospects for treatment [3].

In the last decade, neoadjuvant chemotherapy (NAC) has become a standard of care for TNBC [4]. Pathological complete response (pCR) after NAC occurs in approximately 30% to 50% of cases and is associated with longer disease-free (DFS) and overall survival (OS) [5,6]. For patients not achieving a pCR, the amount of residual disease (RD) can be assessed by determining the residual cancer burden (RCB) index, which can be used to classify patients into several prognostic groups [7,8]. The identification of patients with a poorer prognosis has important implications, as these patients may benefit from second-line treatments, such as adjuvant capecitabine [9,10].

Immune checkpoint inhibitor (ICI) therapies targeting PD-1 [11] and PD-L1 have been shown to be effective against cancers at various sites, and a number of blocking antibodies are currently being tested for use in BC [12,13]. Atezolizumab is used in combination with nab-paclitaxel for the routine treatment of advanced TNBC [14]. In the neoadjuvant setting, pCR rates were found to be higher in TNBC patients treated with durvalumab in addition to NAC than in the placebo arm (53.4% versus 44.2%, respectively) [15]. The emerging biomarkers for ICI efficacy include, principally, (i) programmed cell death-1 (PD1), (ii) its ligand-1 (PD-L1) and (iii) high levels of tumor-infiltrating lymphocytes (TILs) [16,17,18]. In the GeparNuevo trial, a trend towards higher pCR rates in tumors positive for PD-L1 at baseline was observed, which was significant for PD-L1-tumor cells in the durvalumab arm (*p* = 0.045) and for PD-L1-immune cells in the placebo arm (*p* = 0.040), suggesting that high levels of PD-L1 were linked to a stronger response but were not predictive of durvalumab response [15]. Other than these data for pre-NAC PD-L1 expression and the response to ICI, data for PD-L1 expression in tumor cells and/or immune cells in RD are scarce and the prognostic implications are unknown.

It has been suggested that chemotherapy could act as a functional immunotherapy by enhancing the release of tumor-associated antigens capable of triggering an immune response directed against tumor cells [19]. Taking this into account, experts have recommended the systematic assessment of TIL levels in the post-neoadjuvant setting [20]. As changes in the immune microenvironment following NAC could, theoretically, have a significant impact on PD-L1 expression, their assessment is of interest in the neoadjuvant setting [21,22]. We investigated the influence of NAC on TNBC tumors and their micro-immune environment, by analyzing PD-L1 expression in 89 patients with RD after the treatment of TNBC with NAC. Estrogen receptor (ER), progesterone receptor (PR) and human epidermal growth factor receptor 2 (Her2) status and Ki67 levels were also assessed, to decipher the relationships between hormonal and proliferation pathways and PD-L1.

## 2. Materials and Methods

### 2.1. Patients and Tumors

The analysis was performed on 89 patients with triple-negative, invasive, unilateral, non-recurrent, breast carcinoma, stage T1-T3NxM0, treated with NAC at Institut Curie, Paris, between 2002 and 2012 (NEOREP Cohort, CNIL declaration number 1547270). NAC regimens changed over the recruitment period (anthracycline-based regimen or sequential anthracycline-taxane regimens). Surgery was performed four to six weeks after the end of chemotherapy. The study was approved by the Breast Cancer Study Group of Institut Curie and was conducted according to institutional and ethical rules regarding research on tissue specimens and patients. Written informed consent from the patients was not required under French regulations.

### 2.2. Tumor Samples

Tumor samples were reviewed by two specialist pathologists (D.M. and M.L.). The pathological diagnosis was confirmed by initial core needle biopsy (CNB) before treatment. TNBCs were defined as tumors negative for ER, PR and HER2 expression. Cases were considered to be ER- or PR-negative if <1% of the tumor cells expressed ER/PR, in accordance with the American guidelines [23]. HER2 expression was determined by immunohistochemistry (IHC), with scoring according to American Society of Clinical Oncology (ASCO)/College of American Pathologists (CAP) guidelines [24]. Tumor cellularity was defined as the percentage of tumor cells (in situ and invasive) in the specimen slide (biopsy or surgical specimen). Mitotic index was reported per 10 high-power fields (HPF) (1 HPF = 0.301 mm^2^).

We evaluated PD-L1 expression in residual disease as a percentage of tumor cells by membrane staining (PD-L1-TC), and as a percentage of tumor-infiltrating lymphocytes (TILs) by membrane or cytoplasmic staining (PD-L1-IC; relative to total TILs). PD-L1 positivity was defined as expression by ≥1% of the cells, for both tumor and immune cells. For descriptive purposes, we also provide binned data, as follows: PD-L1-TC: 0%, 1%, (1–25%), (25–50%) and ≥50%, and PD-L1-IC: 0%, 1%, (1–5%), (5–10%) and ≥10%. PD-L1-IC immunohistochemical expression was evaluated with antibody: Cell Signaling #13684S (E1L3N^®^) XP^®^ rabbit mAb (Supplementary Figure S1). Immunohistochemical expression for ER, PR, Her2 and Ki67 was evaluated using the following primary antibodies: (i) ER with antibody: Diagomics 10045-10 mAb rabbit IgG SP1, (ii) PR with antibody: Leica NCL-L-PGR312 mAb mouse IgG 16, (iii) Her2 with antibody: Dako A0485 pAb rabbit IgG and (iv) Ki67 with antibody: Dako M7240 mAb mouse IgG1 MIB-1.

### 2.3. Survival Endpoints

Relapse-free survival (RFS) was defined as the time from surgery to death, locoregional recurrence or distant recurrence, whichever occurred first, and overall survival (OS) was defined as the time from surgery to death. Patients for whom none of these events were recorded were censored at the date of last known contact. The cutoff date for survival analysis was 1 February 2019.

### 2.4. Statistical Analysis

The study population was described in terms of frequencies for qualitative variables, or medians and associated ranges for quantitative variables. Chi-squared tests were performed to search for differences between subgroups for each variable (considered significant for *p*-values ≤ 0.05). Continuous variables were compared between groups in Wilcoxon–Mann–Whitney tests for groups of fewer than 30 patients and for variables following multimodal distributions. Student’s t-tests were used in all other cases. Survival probabilities were estimated by the Kaplan–Meier method, and survival curves were compared in log-rank tests. Hazard ratios and their 95% confidence intervals (CI) were calculated with the Cox proportional hazards model. Variables with a *p*-value for the likelihood ratio test of 0.05 or lower in univariate analysis were selected for inclusion in the multivariate analysis. A forward stepwise selection procedure was used to establish the final multivariate model, with a significance threshold of 5%. Data were processed and statistical analyses were carried out with R software version 3.1.2 (www.cran.r-project.org) [14].

## 3. Results

### 3.1. Characteristics of the Patients and Tumors

In total, 1199 patients treated with NAC were included in the institutional cohort of the Institut Curie: 376 had a TNBC, and 231 had RD after completing NAC. The triple-negative tumors for which pCR was achieved were of higher grades, with higher levels of Ki67 and TILs than tumors for which pCR was not achieved (Supplementary Table S1). We retrieved blocks for 122 patients with RD, 89 of which were reviewed and included in this analysis. The patients had a median age of 50.2 years old, and most were premenopausal (*n* = 51, 57%) and had a normal body mass index (BMI) (*n* = 47, 52%). For 69.7% of the tumors, the diagnosis was made at the T2 stage (*n* = 62), mostly with baseline axillary node involvement (*n* = 51, 57.3%). Primary treatment was an anthracycline-taxane regimen in 95% of the patients (Table 1).

All residual tumors remained negative for ER and PR after NAC (*n* = 89). Similarly, HER2 expression was negative in 88 of 89 cases (scored 1+ in 3 patients, 0 in 85 patients), and 2+ in one patient (FISH-negative). No post-NAC switch in BC subtype was found in any of the 89 patients.

#### 3.1.1. Association between Post-NAC PD-L1 Expression and Baseline Clinical and Pathologic Patterns

**Figure 1:**
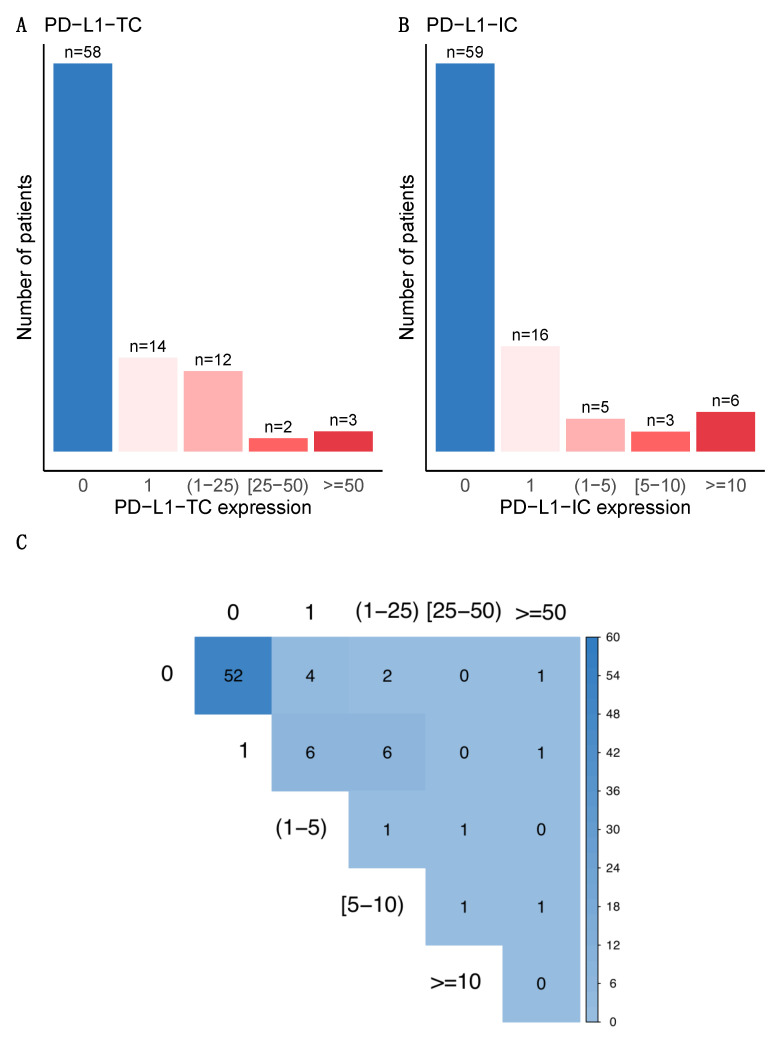
Bar plot distribution of PD-L1 expression after NAC (**A**) on tumor cells and (**B**) on immune cells. (**C**) Correlation plot of PD-L1 expression between tumor cells and immune cells. PD-L1 positivity was defined as expression by ≥1% of the cells (being the 1% included) for both tumor and immune cells. PD-L1 expression were also binned: on tumor cells/0%, 1%, (1–25%), (25–50%) and ≥50%, and on immune cells/0%, 1%, (1–5%), (5–10%) and ≥10%.

**Figure 2:**
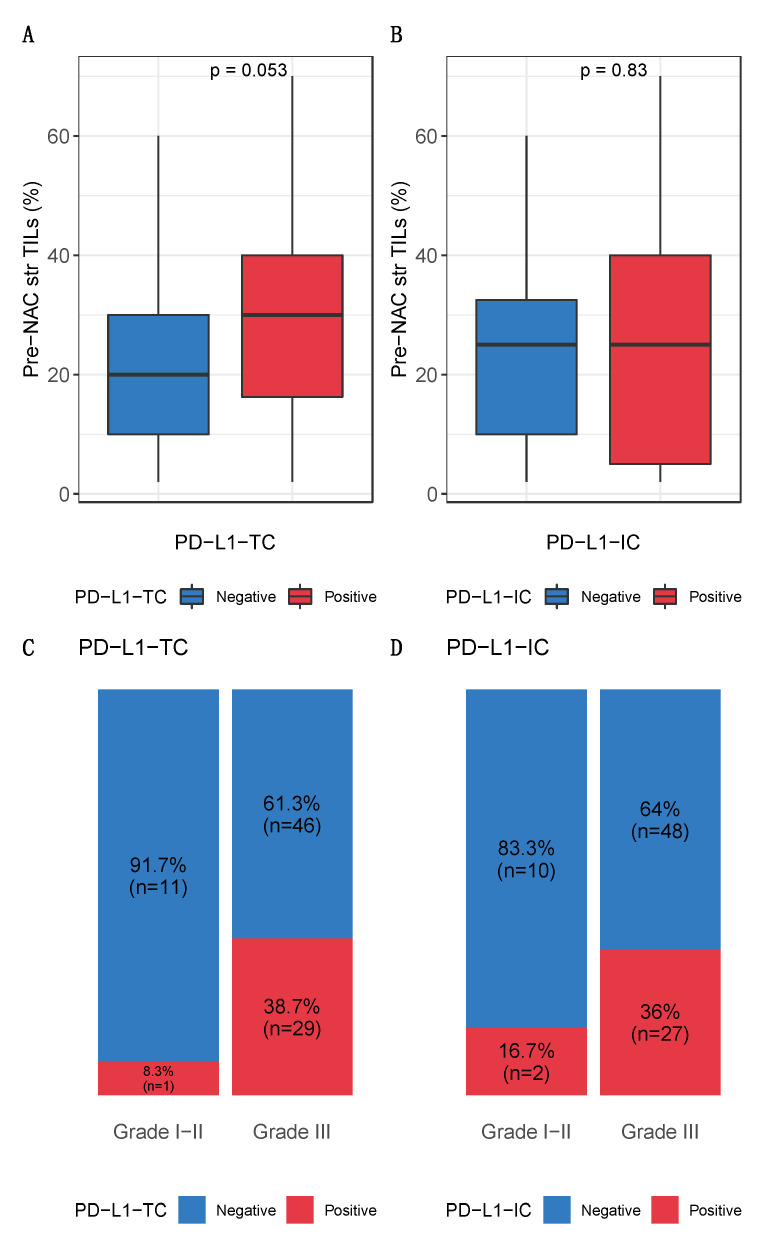
Baseline characteristics of PD-L1-TC and PD-L1-IC expression. Bottom and top bars of the boxplots represent the first and third quartiles respectively, the medium bar is the median, and whiskers extend to 1.5 times the interquartile range. Associations between pre-NAC TILs and (**A**) tumor cells and (**B**) immune cells. Percentage of tumor according to grade, (**C**) by tumor cells and (**D**) by immune cells.

PD-L1 was not expressed (0%) in 65.2% (*n* = 58) of tumors and 66.3% (*n* = 59) of immune cells (Figure 1A,B). PD-L1-TC was associated with PD-L1-IC expression (*p* < 0.001) (Figure 1C). None of the baseline characteristics of the patients were significantly associated with post-NAC PD-L1-TC. High pre-NAC stromal (str) TIL levels were the only tumor characteristic associated with PD-L1 positivity in tumor cells (*p* = 0.05), but no association was found in immune cells (Figure 2, Table 1). The differences in str TIL levels before and after NAC were not associated with PD-L1-TC nor PD-L1-IC positivity (Supplementary Figure S2).

#### 3.1.2. Association between Post-NAC PD-L1 Expression, KI67 and Post-NAC Pathological Patterns

**Figure 3:**
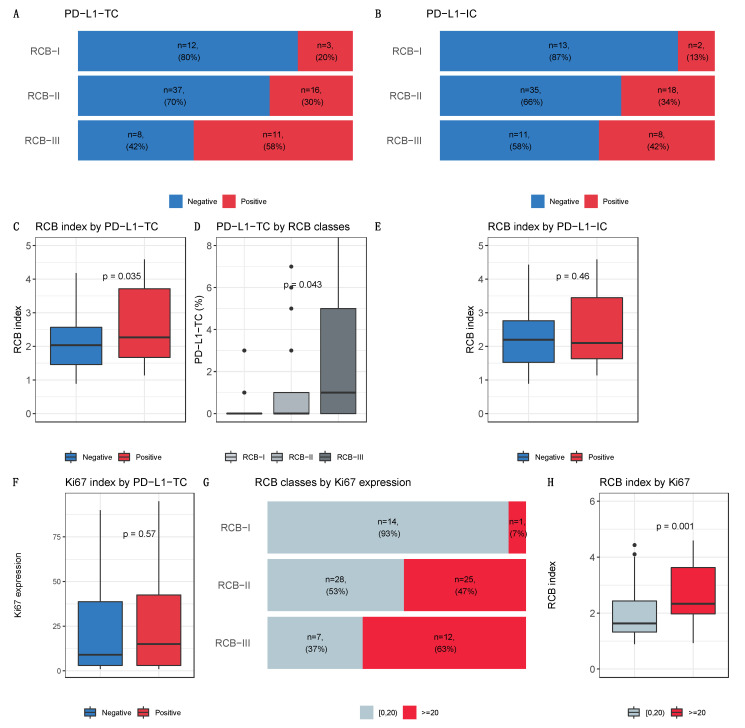
Post-NAC characteristic by PDL1 expression and Ki67 expression. Bottom and top bars of the boxplots represent the first and third quartiles respectively, the medium bar is the median, and whiskers extend to 1.5 times the interquartile range. Percentage of tumor according to RCB classes by PD-L1-TC (5 classes). Percentage of tumors according to RCB classes, (**A**) by PD-L1 tumor cells and (**B**) by PD-L1 immune cells. (**C**) RCB index by PD-L1 tumor cells. (**D**) PD-L1 tumor cells by RCB classes. (**E**) RCB index by PD-L1 immune cells. (**F**) Ki67 index by PD-L1 tumor cells. (**G**) Percentage of tumor according to RCB classes by ki67 expression. (**H**) RCB index by Ki67 expression.

RCB was assessed in 87 of 89 tumors (98%). The proportion of patients within each RCB class was as follows: RCB-I, *n* = 15 (17%), RCB-II, *n* = 53 (61%), and RCB-III, *n* = 19 (22%). PD-L1 expression was significantly associated with a higher RCB, higher post-NAC mitotic index and a trend towards higher tumor cellularity (*p* = 0.06), in tumor cells but not in immune cells (Figure 3A–H, Table S2). PD-L1-TC expression was not associated with Ki67 expression, neither in tumor cells nor in immune cells (*p* = 0.9 and *p* = 1, respectively), but Ki67 was associated with RCB index (Figure 3F–H).

### 3.2. Survival Analyses

**Figure 4:**
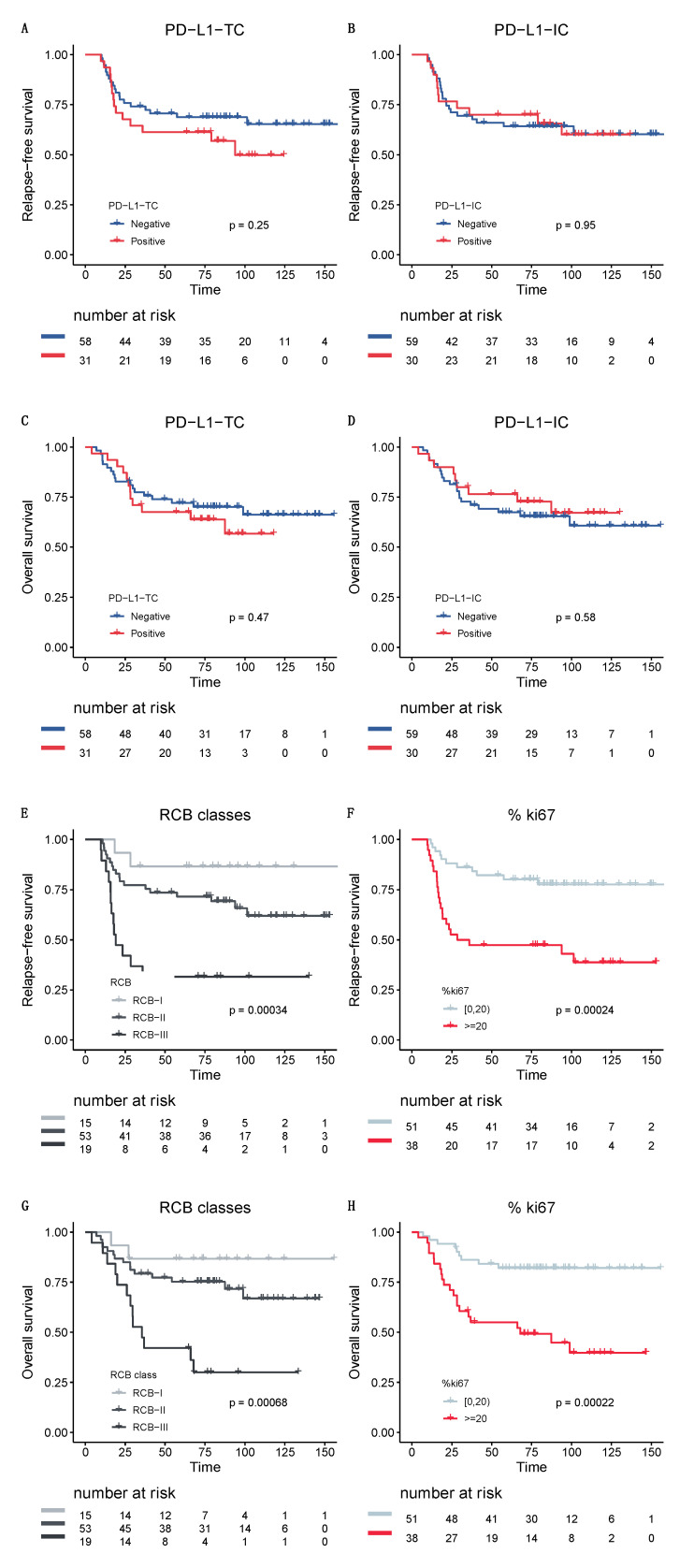
Survival curves. Relapse-free survival (RFS) curves according to (**A**) PD-L1 tumor cells and (**B**) PD-L1 immune cells. Overall survival (OS) curves according to (**C**) PD-L1 tumor cells and (**D**) PD-L1 immune cells. Relapse-free survival curves according to (**E**) RCB classes and (**F**) Ki67 expression. Overall survival curves according to (**G**) RCB classes and (**H**) Ki67 expression.

With a median follow-up of 80 months, 34 patients experienced relapses and 30 patients died. Neither PD-L1-TC nor PD-L1-IC was significantly associated with RFS or OS (Figure 4A–D). By contrast, both RCB and Ki67 were significantly associated with RFS and OS (Figure 4E–H) and were independent predictors of survival in multivariate analysis (RCB index, odds ratio (OR) = 1.7; CI95% (1.25–2.42), *p* < 0.001; post-NAC Ki67 expression, OR = 2.7; CI95% (1.29–5.8), *p* = 0.008, respectively).

## 4. Discussion

In this study, we analyzed PD-L1 expression in tumor and immune cells from residual post-NAC TNBC. We found that PD-L1 expression was significantly associated with a greater tumor burden and aggressive post-NAC tumor characteristics. This study provides new insight into immunity in the post-NAC setting.

First, we observed that the proportions of tumor and immune cells positively stained for PD-L1 (19.1% and 15.7%, respectively) were low. None of the clinical or pathological features of the patients at baseline were associated with post-NAC PD-L1 expression. At least seven meta-analyses, including more than 125 studies, have evaluated PD-L1 expression in BC bulk tumor samples over the last three years (see Supplementary Table S3). The prevalence of PD-L1 positivity ranged from 6.4% to 76.4% [19]. PD-L1 expression was significantly associated with the HR-negative [17,25,26], HER2-positive [25] and TNBC [17,25,26] subtypes, and the levels detected depended on the detection method [19]. To our knowledge, only three studies have analyzed PD-L1 expression on specimens of tumors previously treated with systemic therapy (see Table 2). In the SWOG N0800 neoadjuvant trials, Pelekanou et al. retrospectively analyzed 43 surgical specimens from HER2-negative tumors (TNBC, *n* = 9) by assessing PD-L1 expression with the Dako clone and a 1% cutoff for positivity. They found that PD-L1 expression was relatively stable before and after treatment (43% (52/120) versus 33% (14/43), respectively) [27]. In a study on the same cohort of patients, Li et al. showed that PD-L1 levels in tumor and stromal cells were not significantly affected by treatment (*p* = 0.502 and *p* = 0.655, respectively) [28]. This cohort included both HR-positive and HR-negative BC specimens, and subgroup analyses were not possible due to the small sample size [28]. In a TNBC cohort including 114 patients, Wang et al. found that 37.7% of post-NAC specimens from patients with RD were positive for PD-L1. This rate is higher than that for the cohort reported here, and such discrepancies between studies may be due to the use of a different antibody for PD-L1 expression (EPR19759 clone rather than the E1L3N clone used here).

Second, PD-L1 expression in post-NAC tumor cells was associated with a higher RCB index. High levels of PD-L1 in tumor cells have been linked to clinical and pathological features associated with a poor prognosis in numerous studies. In particular, an association with the presence of lymph node metastasis and high tumor grade has been reported for evaluations of non-pretreated tumors (see Supplementary Table S3). In the pre-NAC setting, Huang et al. published a pooled analysis of eight studies including 1085 patients, and showed that PD-L1 positivity in core specimens before treatment was associated with higher pCR rates (RR = 1.64, 95%CI (0.99; 2.73), *p* = 0.05), but with significant heterogeneity between studies (I^2^ = 79%, P_heterogeneity_ < 0.0001) [26]. No data have been published concerning the association between PD-L1 and response to treatment after NAC. It remains unknown whether the greater RCB observed here for PD-L1-positive tumors is a cause or consequence of the RD. Two mechanisms may be involved: the PD-L1 protein may operate as a suppressor of anti-tumor immune responses [29], preventing immune cells from clearing tumor cells efficiently, or chemotherapy-resistant tumor cells may be more likely to express immunosuppressive factors.

We found no impact of post-NAC PD-L1 expression on oncological outcomes, in terms of either RFS or OS for either tumor cells or immune cells. Studies evaluating the prognostic implications of PD-L1 in BC have reported conflicting results. Some reported a positive association between PD-L1 and survival [30,31,32,33,34], whereas others found a negative association [17,25,26] or no association at all [35]. However, these findings must be interpreted with care, because PD-L1 expression may be differentially regulated on tumor or immune cells [29,36,37], and previous studies have highlighted the importance of studying the expression of this molecule in both types of cell [29]. In a recent large meta-analysis, Matikas and coworkers showed that PD-L1 expression in tumor cells was associated with a shorter DFS and OS, whereas PD-L1 expression in immune cells was associated with a longer DFS (HR = 0.61, 95%CI (0.51–0.73), *p* < 0.001), and with a longer OS (HR = 0.53, 95%CI (0.39–0.73), *p* < 0.001) in the TNBC subgroup (8 studies, *n* = 969) [38]. These studies were performed on bulk tumors, and the prognostic value of PD-L1 may be different in the post-NAC setting. In the only study to date evaluating PD-L1 expression and prognosis in the context of RD, Wang and coworkers found no association between high levels of PD-L1 expression and DFS (*p* = 0.249).

Finally, our study confirms the strong prognostic value of Ki67 expression after NAC, with high levels of expression associated with a poor prognosis. Post-NAC, high Ki67 levels have consistently found to be a predictor of poor DFS across studies [39,40,41,42,43,44,45,46,47,48,49,50,51]. In a matched cohort of 103 BC patients, Jones et al. found post-therapy Ki67 to be the only significant independent prognostic factor for DFS in multivariate analysis (*p* < 0.001), and the strongest prognostic factor for OS (*p* < 0.001) [49]. Ultimately, baseline Ki67 expression is predictive of pCR in patients with RD and persistent highly proliferative disease, but the outcome is poor [51]. The RCB index, first published in 2007, combines pathological findings from both the primary tumor bed and the regional lymph nodes. It has been validated as a predictor of the risk of recurrence in several independent cohorts [8,39] and is now widely used as a primary endpoint in clinical trials [52]. Several studies have suggested that the inclusion of additional factors, such as immunological features [53,54], lymphovascular invasion [55] or post-NAC Ki67 expression [56], could improve its performance. Our results confirm that amidst the candidate post-NAC parameters for improving RCB performance, post-NAC Ki67 expression does indeed have a strong prognostic value, but our results are not consistent with the hypothesis that PD-L1 expression is a strong prognostic marker that could be used to refine estimates of the risk of relapse in the post-NAC setting, despite its association with greater RCB.

This study has several strengths and limitations. First, we provided unprecedented data on PD-L1 expression, using the E1L3N clone in the setting of post-NAC RD in TNBC patients. Our results call for additional evidence on the clinical relevance of such a biomarker for accurately identifying patients in whom it might be a useful theranostic marker. However, our cohort is potentially subject to technical pitfalls, as AFA fixative (a combination of alcohol, formalin, and acetic acid) was used in this historical cohort. It has recently been shown that the use of this fixative decreases antibody binding to PD-L1, and this has led to a switch to formol-only fixation [14]. Similar analyses on more recent paraffin-embedded specimens may therefore be of interest in this context. In addition, as in other studies, the main limitation of our report is the absence of a standardized detection technique and cutoff, as multiple assays and scoring systems exist. A wide range of positivity rates and discordant associations with prognosis have been reported across studies, depending on the antibodies used [26,38]. In our study, PD-L1 expression was assessed with the E1L3N antibody, which has been shown to be more sensitive than 28–8 Dako [57] and the SP142 Ventana Assay [58], but inferior to the SP263 assay [59,60]. Furthermore, different cutoffs are used to define PD-L1 positivity for different tumor types and in clinical trials [21]. The principal cutoffs used were 1% and 5% [38], but cutoffs values ranging from 1% to 50% have been used [26] and several teams use composite scores of staining intensity and the percentage of positive cells [26,38]. A standardized methodology is much needed, to ensure consistency and reproducibility in future PD-L1 studies [21]. Several teams have tried to overcome these discrepancies by using DNA microarrays to analyze PD-L1 mRNA levels [61,62,63].

Our study opens up several pragmatic perspectives. The TIL working group recently suggested that PD-L1 expression should be included in the routine clinical assessment of BC specimens [21]. Our results suggest that (i) further evidence is required to confirm the clinical utility of this marker and its validity in the post-NAC setting, and (ii) standardized guidelines for the assessment of this biomarker should be published before its integration into routine practice. Furthermore, as increasing numbers of patients with TNBC are being treated by NAC, the number of second-line trials in the post-NAC setting is growing [12,13]. Further studies are required to evaluate the proper place of PD-L1 as an immuno-oncological biomarker for selecting patients likely to benefit from ICI in the post-NAC setting.

## 5. Conclusions

In conclusion, our study identified a small subset of patients with TNBCs and RD after NAC displaying PD-L1 expression in the context of a higher post-NAC tumor burden. As RCB is associated with a higher risk of relapse, and as these patients could theoretically respond to ICI [16], they could be invited to participate in second-line treatment trials of immunotherapy after NAC.

## Figures and Tables

**Table 1:** Patient characteristics among TNBC according to PD-L1-TC and PD-L1-IC expression.

			PDL-L1-TC			PDL-L1-IC		
Characteristics	Class	Overall	Negative (0%)	Positive (≥1%)	*p*	Negative (0%)	Positive (≥1%)	*p*
n=		89	58	31		59	30	
**Baseline Characteristics**								
Age		50.20 [39.40, 57.90]	49.03 (10.87)	49.16 (11.23)	0.960	48.94 (10.54)	49.34 (11.85)	0.871
Family history	No	68 (76.4)	44 (75.9)	24 (77.4)	1.000	46 (78.0)	22 (73.3)	0.824
	Yes	21 (23.6)	14 (24.1)	7 (22.6)		13 (22.0)	8 (26.7)	
Menopausal status	Premenopausal	51 (57.3)	33 (56.9)	18 (58.1)	1.000	34 (57.6)	17 (56.7)	1.000
	Postmenopausal	38 (42.7)	25 (43.1)	13 (41.9)		25 (42.4)	13 (43.3)	
BMI classes	18.5–24.9	47 (52.8)	34 (58.6)	13 (41.9)	0.266	34 (57.6)	13 (43.3)	0.221
	<18.5	2 (2.2)	2 (3.4)	0 (0.0)		2 (3.4)	0 (0.0)	
	25–29.9	22 (24.7)	12 (20.7)	10 (32.3)		11 (18.6)	11 (36.7)	
	>=30	18 (20.2)	10 (17.2)	8 (25.8)		12 (20.3)	6 (20.0)	
Smoking status	Never	61 (71.8)	42 (75.0)	19 (65.5)	0.452	42 (73.7)	19 (67.9)	0.366
	Current	12 (14.1)	8 (14.3)	4 (13.8)		9 (15.8)	3 (10.7)	
	Former	12 (14.1)	6 (10.7)	6 (20.7)		6 (10.5)	6 (21.4)	
Comorbidity	No	35 (45.5)	25 (49.0)	10 (38.5)	0.524	27 (51.9)	8 (32.0)	0.162
	Yes	42 (54.5)	26 (51.0)	16 (61.5)		25 (48.1)	17 (68.0)	
Clinicar T stage	T0–T1	6 (6.7)	2 (3.4)	4 (12.9)	0.077	4 (6.8)	2 (6.7)	0.257
	T2	62 (69.7)	39 (67.2)	23 (74.2)		38 (64.4)	24 (80.0)	
	T3–T4	21 (23.6)	17 (29.3)	4 (12.9)		17 (28.8)	4 (13.3)	
Clinical N stage	N0	38 (42.7)	25 (43.1)	13 (41.9)	1.000	26 (44.1)	12 (40.0)	0.889
	N1–N2–N3	51 (57.3)	33 (56.9)	18 (58.1)		33 (55.9)	18 (60.0)	
SBR grade	Grade I-II	12 (13.8)	11 (19.3)	1 (3.3)	0.084	10 (17.2)	2 (6.9)	0.323
	Grade III	75 (86.2)	46 (80.7)	29 (96.7)		48 (82.8)	27 (93.1)	
Mitotic index		32.62 (25.80)	23.00 [11.00, 40.25]	35.50 [14.75, 52.75]	0.165	24.00 [11.00, 41.00]	30.00 [14.00, 52.00]	0.283
Mitotic index class	[0,20)	34 (41.5)	24 (44.4)	10 (35.7)	0.600	24 (43.6)	10 (37.0)	0.740
	>=20	48 (58.5)	30 (55.6)	18 (64.3)		31 (56.4)	17 (63.0)	
Ductal carcinoma in situ	No	83 (93.3)	53 (91.4)	30 (96.8)	0.601	55 (93.2)	28 (93.3)	1.000
	Yes	6 (6.7)	5 (8.6)	1 (3.2)		4 (6.8)	2 (6.7)	
% stromal lymphocytes		25.00 [10.00, 40.00]	20.00 [10.00, 30.00]	30.00 [16.25, 40.00]	0.053	25.00 [10.00, 32.50]	25.00 [5.00, 40.00]	0.823
BC surgery	Lumpectomy	59 (66.3)	41 (70.7)	18 (58.1)	0.334	42 (71.2)	17 (56.7)	0.257
	Mastectomy	30 (33.7)	17 (29.3)	13 (41.9)		17 (28.8)	13 (43.3)	
NAC regimen	anthra-taxans	85 (95.5)	55 (94.8)	30 (96.8)	0.761	56 (94.9)	29 (96.7)	0.773
	anthra	3 (3.4)	2 (3.4)	1 (3.2)		2 (3.4)	1 (3.3)	
	taxanes	1 (1.1)	1 (1.7)	0 (0.0)		1 (1.7)	0 (0.0)	
**Post-NAC characteristics**								
ypN stage	0	53 (59.6)	35 (60.3)	18 (58.1)	0.109	33 (55.9)	20 (66.7)	0.065
	[1-3]	22 (24.7)	17 (29.3)	5 (16.1)		19 (32.2)	3 (10.0)	
	[4-9]	13 (14.6)	5 (8.6)	8 (25.8)		6 (10.2)	7 (23.3)	
	10 and more	1 (1.1)	1 (1.7)	0 (0.0)		1 (1.7)	0 (0.0)	
RCB index		2.12 [1.54, 2.98]	2.03 [1.45, 2.56]	2.27 [1.67, 3.72]	0.035	2.19 [1.52, 2.76]	2.10 [1.63, 3.45]	0.456
RCB classes	RCB-I	15 (17.2)	12 (21.1)	3 (10.0)	0.040	13 (22.0)	2 (7.1)	0.185
		53 (60.9)	37 (64.9)	16 (53.3)		35 (59.3)	18 (64.3)	
	RCB-III	19 (21.8)	8 (14.0)	11 (36.7)		11 (18.6)	8 (28.6)	
KI67 status	[0,20)	51 (57.3)	34 (58.6)	17 (54.8)	0.905	34 (57.6)	17 (56.7)	1.000
	>=20	38 (42.7)	24 (41.4)	14 (45.2)		25 (42.4)	13 (43.3)	
Mitotic index		31.28 (40.93)	15.00 [0.00, 47.00]	25.00 [3.00, 70.00]	0.036	17.00 [0.00, 50.00]	14.00 [2.50, 51.50]	0.541
Mitotic index class	[0,20)	43 (55.1)	29 (55.8)	14 (53.8)	1.000	28 (53.8)	15 (57.7)	0.936
	>=20	35 (44.9)	23 (44.2)	12 (46.2)		24 (46.2)	11 (42.3)	
% stromal lymphocytes		15.00 [10.00, 30.00]	15.00 [10.00, 30.00]	20.00 [6.25, 30.00]	0.860	15.00 [10.00, 30.00]	22.50 [5.00, 30.00]	0.850
Lymphovascular invasion	No	54 (70.1)	32 (65.3)	22 (78.6)	0.335	32 (65.3)	22 (78.6)	0.335
	Yes	23 (29.9)	17 (34.7)	6 (21.4)		17 (34.7)	6 (21.4)	

Abbreviations: TC = tumor cells; IC = immune cells; BMI = body mass index; T = tumor; N = node; SBR = grade Scarff-Bloom and Richardson; NAC = neoadjuvant chemotherapy; anthra-taxans = anthracyclines and taxanes; anthra = anthracyclines; RCB = Residual Cancer Burden. The “n” denotes the number of patients. In case of categorical variables, percentages are expressed between brackets. In case of continuous variables, mean value is reported. In case of nonnormal continuous variables, median value is reported, with interquartile range between brackets. Missing data: Smoking status, n = 4; Comorbidity, n = 12; SBR grade, n = 2; Mitotic index, n = 3; Mitotic index class, n = 7; % stromal lymphocytes, n = 2; RCB index, n = 2; RCB classes, n = 2; Mitotic index post-NAC, n = 7; Mitotic index class post-NAC, n = 11; % stromal lymphocytes post-NAC, n = 2; Lymphovascular invasion post-NAC, n = 12. NB = Pre-NAC Ki67 not displayed in the table due to the high among of missing values (n = 81). Among the patients with pre-NAC Ki67 available, all values were 20% or higher.

**Table 2:** Studies on PD-L1 expression in BC RD.

First Author	Journal	Year	Country	*n* Patients (RD)	Detection Technique (IHC Clone)	Cut-Off	PDL-1 Prevalence in CNB	PDL1 and pCR	PDL-1 Prevalence in RD	PDL1 and DFS	PDL1 and OS
Pelekanou (SWOG S0800) [64]	Breast cancer research	2017	USA	58 (Her2-negative)	RD: SP142 and 22C3 Dako combined CNB: E1L3N	QIF = 500	21/41 (51.0%)	*p* = 0.018	10 (17.2%)	-	-
Pelekanou (SWOG S0800) [27]	Molecular cancer therapeutics	2018	USA	43 (Her2-negative) out of which 9 TNBC	22C3 Dako	QIF = 500	52/120 (43%)	higher pCR rates immune + tumor c. (*p* = 0.008) sole tumor c. NS (*p* = 0.10)	14 (33%)	- (in CNB NS *p* = 0.14)	- (in CNB NS *p* = 0.64)
Wang [25]	Journal of Breast Cancer	2018	China	114 TNBC	EPR19759	H-score of 100	Tumor and/or Immune c. 48 (32.4%)	HR = 1.34 (0.54; 3.30) *p* = 0.53	43 (37.7%)	Tumor/Immune c. NS; HR = 1.421 (0.78; 2.59) *p* = 0.249	-
Li (SWOG S0800) [28]	Journal for Immunotherapy of Cancer	2019	USA	60 (Her2-negative)	22C3 Dako	1%	-	NS in tumor c. (*p* = 0.1578) and immune c. (*p* = 0.0722)	-	-	-
Loibl (GeparNuevo study) [15]	Annals of Oncology	2019	Germany	174 TNBC	SP263 Ventana	1%	Tumor and/or Immune c. 138/158 (87.3%)	Durvalumab group 47/88 (53.4%) Placebo group 38/86 (44.2%)	-	-	-
Srivastava [65]	Journal of Family Medicine and Primary Care	2020	India	30	Abcam NA	H-score of 100	11 (36.7%)	-	4 (13.3%)	-	-
Grandal	Cancers	2020	France	89 TNBC	E1L3N	1%	-	-	Tumor c. *n* = 17 (19.1%) Immune c. *n* = 14 (15.7%)	Tumor c. NS (*p* = 0.38) Immune c. NS (*p* = 0.23)	Tumor c. NS (*p* = 0.48) Immune c. NS (*p* = 0.46)

Abbreviations: residual disease (RD), core needle biopsy (CNB), breast cancer (BC), cells (c.), triple-negative breast cancer (TNBC), hazard ratio (HR), confidence interval (CI), not significant (NS), relative risk (RR), pathological complete response (pCR).

## Data Availability

Data available on request due to privacy/ethical restrictions. The data that support the findings of this study are available on request from the corresponding author, [FR]. The data are not publicly available because they contain information that could compromise the privacy of research participants.

## Supplementary Materials

The following are available online at https://www.mdpi.com/2072-6694/13/4/746/s1, Figure S1: Immunohistochemical analysis of PD-L1 in surgical specimens (Cell Signaling #13684S (E1L3N®) XP® rabbit mAb) staining, Figure S2: Pre-NAC and post-NAC stromal immune infiltration rates in the whole population and by BRCA status, Table S1: Patient characteristics among the whole population and by pCR status, Table S2: Association of clinical and pathological pre and post-NAC parameters with PD-L1-TC expressio after univariate and multivariate analysis in the whole population, Table S3: Studies on PD-L1 expression in BC RD.

## References

[1] Le Cancer du Sein-Les Cancers Les Plus Fréquents. https://www.e-cancer.fr/Professionnels-de-sante/Les-chiffres-du-cancer-en-France/Epidemiologie-des-cancers/Les-cancers-les-plus-frequents/Cancer-du-sein.

[2] Foulkes W.D., Smith I.E., Reis-Filho J.S. (2010). Triple-negative breast cancer. N. Engl. J. Med..

[3] Lebert J.M., Lester R., Powell E., Seal M., McCarthy J. (2018). Advances in the systemic treatment of triple-negative breast cancer. Curr. Oncol..

[4] Pusztai L., Foldi J., Dhawan A., DiGiovanna M.P., Mamounas E.P. (2019). Changing frameworks in treatment sequencing of triple-negative and HER2-positive, early-stage breast cancers. Lancet Oncol..

[5] Balko J.M., Giltnane J.M., Wang K., Schwarz L.J., Young C.D., Cook R.S., Owens P., Sanders M.E., Kuba M.G., Sánchez V. (2014). Molecular profiling of the residual disease of triple-negative breast cancers after neoadjuvant chemotherapy identifies actionable therapeutic targets. Cancer Discov..

[6] Cortazar P., Zhang L., Untch M., Mehta K., Costantino J.P., Wolmark N., Bonnefoi H., Cameron D., Gianni L., Valagussa P. (2014). Pathological complete response and long-term clinical benefit in breast cancer: The CTNeoBC pooled analysis. Lancet Lond. Engl..

[7] Symmans W.F., Peintinger F., Hatzis C., Rajan R., Kuerer H., Valero V., Assad L., Poniecka A., Hennessy B., Green M. (2007). Measurement of residual breast cancer burden to predict survival after neoadjuvant chemotherapy. J. Clin. Oncol. Off. J. Am. Soc. Clin. Oncol..

[8] Hamy A.-S., Darrigues L., Laas E., De Croze D., Topciu L., Lam G.-T., Evrevin C., Rozette S., Laot L., Lerebours F. (2020). Prognostic value of the Residual Cancer Burden index according to breast cancer subtype: Validation on a cohort of BC patients treated by neoadjuvant chemotherapy. PLoS ONE.

[9] Masuda N., Lee S.-J., Ohtani S., Im Y.-H., Lee E.-S., Yokota I., Kuroi K., Im S.-A., Park B.-W., Kim S.-B. (2017). Adjuvant capecitabine for breast cancer after preoperative chemotherapy. N. Engl. J. Med..

[10] von Minckwitz G., Huang C.-S., Mano M.S., Loibl S., Mamounas E.P., Untch M., Wolmark N., Rastogi P., Schneeweiss A., Redondo A. (2019). Trastuzumab emtansine for residual invasive HER2-positive breast cancer. N. Engl. J. Med..

[11] Nanda R., Liu M.C., Yau C., Shatsky R., Pusztai L., Wallace A., Chien A.J., Forero-Torres A., Ellis E., Han H. (2020). Effect of pembrolizumab plus neoadjuvant chemotherapy on pathologic complete response in women with early-stage breast cancer: An analysis of the ongoing phase 2 adaptively randomized I-SPY2 trial. JAMA Oncol..

[12] Neoadjuvant Study of Abemaciclib, Durvalumab, and an Aromatase Inhibitor Early Stage Breast Cancer-Full Text View-ClinicalTrials.gov. https://clinicaltrials.gov/ct2/show/NCT04088032.

[13] Merck Sharp & Dohme Corp (2020). A Randomized, Double-Blind, Phase III Study of Pembrolizumab Versus Placebo in Combination with Neoadjuvant Chemotherapy and Adjuvant Endocrine Therapy for the Treatment of High-Risk Early-Stage Estrogen Receptor-Positive, Human Epidermal Growth Factor Receptor 2-Negative (ER+/HER2-) Breast Cancer (KEYNOTE-756).

[14] Schmid P., Adams S., Rugo H.S., Schneeweiss A., Barrios C.H., Iwata H., Diéras V., Hegg R., Im S.-A., Shaw Wright G. (2018). Atezolizumab and nab-paclitaxel in advanced triple-negative breast cancer. N. Engl. J. Med..

[15] Loibl S., Untch M., Burchardi N., Huober J., Sinn B.V., Blohmer J.-U., Grischke E.-M., Furlanetto J., Tesch H., Hanusch C. (2019). A randomised phase II study investigating durvalumab in addition to an anthracycline taxane-based neoadjuvant therapy in early triple-negative breast cancer: Clinical results and biomarker analysis of GeparNuevo study. Ann. Oncol..

[16] Emens L.A. (2018). Breast cancer immunotherapy: Facts and hopes. Clin. Cancer Res. Off. J. Am. Assoc. Cancer Res..

[17] Zhang M., Sun H., Zhao S., Wang Y., Pu H., Wang Y., Zhang Q. (2017). Expression of PD-L1 and prognosis in breast cancer: A meta-analysis. Oncotarget.

[18] Gandini S., Massi D., Mandalà M. (2016). PD-L1 expression in cancer patients receiving anti PD-1/PD-L1 antibodies: A systematic review and meta-analysis. Crit. Rev. Oncol. Hematol..

[19] Wolff A.C., Hammond M.E.H., Schwartz J.N., Hagerty K.L., Allred D.C., Cote R.J., Dowsett M., Fitzgibbons P.L., Hanna W.M., Langer A. (2007). American Society of Clinical Oncology/College of American Pathologists guideline recommendations for human epidermal growth factor receptor 2 testing in breast cancer. Arch. Pathol. Lab. Med..

[20] Dieci M.V., Radosevic-Robin N., Fineberg S., van den Eynden G., Ternes N., Penault-Llorca F., Pruneri G., D’Alfonso T.M., Demaria S., Castaneda C. (2018). Update on tumor-infiltrating lymphocytes (TILs) in breast cancer, including recommendations to assess TILs in residual disease after neoadjuvant therapy and in carcinoma in situ: A report of the International Immuno-Oncology Biomarker Working Group on Breast Cancer. Semin. Cancer Biol..

[21] Gonzalez-Ericsson P.I., Stovgaard E.S., Sua L.F., Reisenbichler E., Kos Z., Carter J.M., Michiels S., Le Quesne J., Nielsen T.O., Laenkholm A.-V. (2020). The path to a better biomarker: Application of a risk management framework for the implementation of PD-L1 and TILs as immuno-oncology biomarkers in breast cancer clinical trials and daily practice. J. Pathol..

[22] Axelrod M.L., Nixon M.J., Gonzalez-Ericsson P.I., Bergman R.E., Pilkinton M.A., McDonnell W.J., Sanchez V., Opalenik S.R., Loi S., Zhou J. (2020). Changes in peripheral and local tumor immunity after neoadjuvant chemotherapy reshape clinical outcomes in patients with breast cancer. Clin. Cancer Res..

[23] Balaton A.J., Doussal V.L., Arnould L., Barlier C., Bellocq J.P., Ettore F., Fiche M., Jacquemier J., Grogan G.M., Mathieu M.C. (2008). Recommandations pour l’évaluation immunohistochimique des récepteurs hormonaux sur coupes en paraffine dans les carcinomes mammaires Mise à jour 1999. https://www.em-consulte.com/article/88258/recommandations-pour-l-evaluation-immunohistochimi.

[24] Li S., Chen L., Jiang J. (2019). Role of programmed cell death ligand-1 expression on prognostic and overall survival of breast cancer. Medicine.

[25] Wang C., Zhu H., Zhou Y., Mao F., Lin Y., Pan B., Zhang X., Xu Q., Huang X., Sun Q. (2017). Prognostic value of PD-L1 in breast cancer: A meta-analysis. Breast J..

[26] Huang W., Ran R., Shao B., Li H. (2019). Prognostic and clinicopathological value of PD-L1 expression in primary breast cancer: A meta-analysis. Breast Cancer Res. Treat..

[27] Pelekanou V., Barlow W.E., Nahleh Z.A., Wasserman B., Lo Y.-C., von Wahlde M.-K., Hayes D., Hortobagyi G.N., Gralow J., Tripathy D. (2018). Tumor infiltrating lymphocytes and PD-L1 expression in pre- and post-treatment breast cancers in the SWOG S0800 Phase II neoadjuvant chemotherapy trial. Mol. Cancer Ther..

[28] Li X., Warren S., Pelekanou V., Wali V., Cesano A., Liu M., Danaher P., Elliott N., Nahleh Z.A., Hayes D.F. (2019). Immune profiling of pre- and post-treatment breast cancer tissues from the SWOG S0800 neoadjuvant trial. J. Immunother. Cancer.

[29] Noguchi T., Ward J.P., Gubin M.M., Arthur C.D., Lee S.H., Hundal J., Selby M.J., Graziano R.F., Mardis E.R., Korman A.J. (2017). Temporally distinct PD-L1 expression by tumor and host cells contributes to immune escape. Cancer Immunol. Res..

[30] Bae S.B., Cho H.D., Oh M.-H., Lee J.-H., Jang S.-H., Hong S.A., Cho J., Kim S.Y., Han S.W., Lee J.E. (2016). Expression of programmed death receptor ligand 1 with high tumor-infiltrating lymphocytes is associated with better prognosis in breast cancer. J. Breast Cancer.

[31] Beckers R.K., Selinger C.I., Vilain R., Madore J., Wilmott J.S., Harvey K., Holliday A., Cooper C.L., Robbins E., Gillett D. (2016). Programmed death ligand 1 expression in triple-negative breast cancer is associated with tumour-infiltrating lymphocytes and improved outcome. Histopathology.

[32] Baptista M.Z., Sarian L.O., Derchain S.F.M., Pinto G.A., Vassallo J. (2016). Prognostic significance of PD-L1 and PD-L2 in breast cancer. Hum. Pathol..

[33] Sun W.Y., Lee Y.K., Koo J.S. (2016). Expression of PD-L1 in triple-negative breast cancer based on different immunohistochemical antibodies. J. Transl. Med..

[34] Tsang J.Y.S., Au W.-L., Lo K.-Y., Ni Y.-B., Hlaing T., Hu J., Chan S.-K., Chan K.-F., Cheung S.-Y., Tse G.M. (2017). PD-L1 expression and tumor infiltrating PD-1+ lymphocytes associated with outcome in HER2+ breast cancer patients. Breast Cancer Res. Treat..

[35] Lotfinejad P., Asghari Jafarabadi M., Abdoli Shadbad M., Kazemi T., Pashazadeh F., Sandoghchian Shotorbani S., Jadidi Niaragh F., Baghbanzadeh A., Vahed N., Silvestris N. (2020). Prognostic role and clinical significance of tumor-infiltrating lymphocyte (TIL) and programmed death ligand 1 (PD-L1) expression in triple-negative breast cancer (TNBC): A systematic review and meta-analysis study. Diagnostics.

[36] Shin D.S., Zaretsky J.M., Escuin-Ordinas H., Garcia-Diaz A., Hu-Lieskovan S., Kalbasi A., Grasso C.S., Hugo W., Sandoval S., Torrejon D.Y. (2017). Primary resistance to PD-1 blockade mediated by JAK1/2 mutations. Cancer Discov..

[37] Sun C., Mezzadra R., Schumacher T.N. (2018). Regulation and function of the PD-L1 checkpoint. Immunity.

[38] Matikas A., Zerdes I., Lövrot J., Richard F., Sotiriou C., Bergh J., Valachis A., Foukakis T. (2019). Prognostic implications of PD-L1 expression in breast cancer: Systematic review and meta-analysis of immunohistochemistry and pooled analysis of transcriptomic data. Clin. Cancer Res. Off. J. Am. Assoc. Cancer Res..

[39] Miglietta L., Morabito F., Provinciali N., Canobbio L., Meszaros P., Naso C., Murialdo R., Boitano M., Salvi S., Ferrarini M. (2013). A prognostic model based on combining estrogen receptor expression and Ki-67 value after neoadjuvant chemotherapy predicts clinical outcome in locally advanced breast cancer: Extension and analysis of a previously reported cohort of patients. Eur. J. Surg. Oncol. EJSO.

[40] Tokuda E., Horimoto Y., Arakawa A., Himuro T., Senuma K., Nakai K., Saito M. (2017). Differences in Ki67 expressions between pre- and post-neoadjuvant chemotherapy specimens might predict early recurrence of breast cancer. Hum. Pathol..

[41] Matsubara N., Mukai H., Masumoto M., Sasaki M., Naito Y., Fujii S., Wada N. (2014). Survival outcome and reduction rate of Ki-67 between pre- and post-neoadjuvant chemotherapy in breast cancer patients with non-pCR. Breast Cancer Res. Treat..

[42] Montagna E., Bagnardi V., Viale G., Rotmensz N., Sporchia A., Cancello G., Balduzzi A., Galimberti V., Veronesi P., Luini A. (2015). Changes in PgR and Ki-67 in residual tumour and outcome of breast cancer patients treated with neoadjuvant chemotherapy. Ann. Oncol..

[43] Lee H.-C., Ko H., Seol H., Noh D.-Y., Han W., Kim T.-Y., Im S.-A., Park I.A. (2013). Expression of immunohistochemical markers before and after neoadjuvant chemotherapy in breast carcinoma, and their use as predictors of response. J. Breast Cancer.

[44] Faneyte I.F., Schrama J.G., Peterse J.L., Remijnse P.L., Rodenhuis S., van de Vijver M.J. (2003). Breast cancer response to neoadjuvant chemotherapy: Predictive markers and relation with outcome. Br. J. Cancer.

[45] Enomoto Y., Morimoto T., Nishimukai A., Higuchi T., Yanai A., Miyagawa Y., Murase K., Imamura M., Takatsuka Y., Nomura T. (2016). Impact of biomarker changes during neoadjuvant chemotherapy for clinical response in patients with residual breast cancers. Int. J. Clin. Oncol..

[46] Chen X., He C., Han D., Zhou M., Wang Q., Tian J., Li L., Xu F., Zhou E., Yang K. (2017). The predictive value of Ki-67 before neoadjuvant chemotherapy for breast cancer: A systematic review and meta-analysis. Future Oncol. Lond. Engl..

[47] Sánchez-Muñoz A., Plata-Fernández Y.M., Fernández M., Jaén-Morago A., Fernández-Navarro M., de la Torre-Cabrera C., Ramirez-Tortosa C., Lomas-Garrido M., Llácer C., Navarro-Perez V. (2013). The role of immunohistochemistry in breast cancer patients treated with neoadjuvant chemotherapy: An old tool with an enduring prognostic value. Clin. Breast Cancer.

[48] Ács B., Zámbó V., Vízkeleti L., Szász A.M., Madaras L., Szentmártoni G., Tőkés T., Molnár B.Á., Molnár I.A., Vári-Kakas S. (2017). Ki-67 as a controversial predictive and prognostic marker in breast cancer patients treated with neoadjuvant chemotherapy. Diagn. Pathol..

[49] Jones R.L., Salter J., A’Hern R., Nerurkar A., Parton M., Reis-Filho J.S., Smith I.E., Dowsett M. (2009). The prognostic significance of Ki67 before and after neoadjuvant chemotherapy in breast cancer. Breast Cancer Res. Treat..

[50] Tanei T., Shimomura A., Shimazu K., Nakayama T., Kim S.J., Iwamoto T., Tamaki Y., Noguchi S. (2011). Prognostic significance of Ki67 index after neoadjuvant chemotherapy in breast cancer. Eur. J. Surg. Oncol..

[51] Yerushalmi R., Woods R., Ravdin P.M., Hayes M.M., Gelmon K.A. (2010). Ki67 in breast cancer: Prognostic and predictive potential. Lancet Oncol..

[52] Provenzano E., Bossuyt V., Viale G., Cameron D., Badve S., Denkert C., MacGrogan G., Penault-Llorca F., Boughey J., Curigliano G. (2015). Standardization of pathologic evaluation and reporting of postneoadjuvant specimens in clinical trials of breast cancer: Recommendations from an international working group. Mod. Pathol..

[53] Pinard C., Debled M., Ben Rejeb H., Velasco V., Tunon de Lara C., Hoppe S., Richard E., Brouste V., Bonnefoi H., MacGrogan G. (2020). Residual cancer burden index and tumor-infiltrating lymphocyte subtypes in triple-negative breast cancer after neoadjuvant chemotherapy. Breast Cancer Res. Treat..

[54] Hamy A.-S., Pierga J.-Y., Sabaila A., Laas E., Bonsang-Kitzis H., Laurent C., Vincent-Salomon A., Cottu P., Lerebours F., Rouzier R. (2017). Stromal lymphocyte infiltration after neoadjuvant chemotherapy is associated with aggressive residual disease and lower disease-free survival in HER2-positive breast cancer. Ann. Oncol. Off. J. Eur. Soc. Med. Oncol..

[55] Hamy A.-S., Lam G.-T., Laas E., Darrigues L., Balezeau T., Guerin J., Livartowski A., Sadacca B., Pierga J.-Y., Vincent-Salomon A. (2018). Lymphovascular invasion after neoadjuvant chemotherapy is strongly associated with poor prognosis in breast carcinoma. Breast Cancer Res. Treat..

[56] Miglietta F., Dieci M.V., Tsvetkova V., Griguolo G., Vernaci G., Menichetti A., Faggioni G., Giarratano T., Mioranza E., Genovesi E. (2020). Validation of residual proliferative cancer burden as a predictor of long-term outcome following neoadjuvant chemotherapy in patients with hormone receptor-positive/human epidermal growth receptor 2-negative breast cancer. Oncologist.

[57] Cogswell J., Inzunza H.D., Wu Q., Feder J.N., Mintier G., Novotny J., Cardona D.M. (2017). An analytical comparison of Dako 28-8 PharmDx Assay and an E1L3N laboratory-developed test in the immunohistochemical detection of programmed death-ligand 1. Mol. Diagn. Ther..

[58] Schats K.A., Van Vré E.A., De Schepper S., Boeckx C., Schrijvers D.M., Waelput W., Fransen E., Vanden Bempt I., Neyns B., De Meester I. (2017). Validated programmed cell death ligand 1 immunohistochemistry assays (E1L3N and SP142) reveal similar immune cell staining patterns in melanoma when using the same sensitive detection system. Histopathology.

[59] Ahn S., Lee Y., Kim J.-W., Lee J.-C., Hwang J.-H., Yoon Y.-S., Cho J.Y., Han H.-S., Choi Y., Kim H. (2019). Programmed cell death ligand-1 (PD-L1) expression in extrahepatic biliary tract cancers: A comparative study using 22C3, SP263 and E1L3N anti-PD-L1 antibodies. Histopathology.

[60] Smith J., Robida M.D., Acosta K., Vennapusa B., Mistry A., Martin G., Yates A., Hnatyszyn H.J. (2016). Quantitative and qualitative characterization of two PD-L1 clones: SP263 and E1L3N. Diagn. Pathol..

[61] Bertucci F., Finetti P., Colpaert C., Mamessier E., Parizel M., Dirix L., Viens P., Birnbaum D., van Laere S. (2015). PDL1 expression in inflammatory breast cancer is frequent and predicts for the pathological response to chemotherapy. Oncotarget.

[62] Sabatier R., Finetti P., Mamessier E., Adelaide J., Chaffanet M., Ali H.R., Viens P., Caldas C., Birnbaum D., Bertucci F. (2015). Prognostic and predictive value of PDL1 expression in breast cancer. Oncotarget.

[63] Mittendorf E.A., Philips A.V., Meric-Bernstam F., Qiao N., Wu Y., Harrington S., Su X., Wang Y., Gonzalez-Angulo A.M., Akcakanat A. (2014). PD-L1 expression in triple-negative breast cancer. Cancer Immunol. Res..

[64] Pelekanou V., Carvajal-Hausdorf D.E., Altan M., Wasserman B., Carvajal-Hausdorf C., Wimberly H., Brown J., Lannin D., Pusztai L., Rimm D.L. (2017). Effect of Neoadjuvant Chemotherapy on Tumor-Infiltrating Lymphocytes and PD-L1 Expression in Breast Cancer and Its Clinical Significance. Breast Cancer Res..

[65] Srivastava V., Akshay B.R., Kumari S., Meena R.N., Khanna R. (2020). Effect of Neoadjuvant Chemotherapy (NAC) on Programmed Cell Death Ligand (PD-L1) in Patients of Carcinoma Breast: A Prospective Study in Indian Tertiary Care Setting. J. Fam. Med. Prim. Care.

