# Supplementary material for: PD-L1 Expression after Neoadjuvant Chemotherapy in Triple-Negative Breast Cancers Is Associated with Aggressive Residual Disease, Suggesting a Potential for Immunotherapy
Source: Cancers (Basel). 2021 Feb 11;13(4):746. doi: 10.3390/cancers13040746 (PMC7916886; doi:10.3390/cancers13040746)
Supplement: Supplementary file 1 [file cancers-13-00746-s001.pdf]

SUPPLEMENTARY DATA

Supplementary tables

Table S1: Patient characteristics among the whole population and by pCR status.

Missing data: Family history, n=1; Menopausal status, n=4; BMI classes, n=1; Smoking status, n=71; Comorbidity, n=56; Nuclear ER staining (%), n=20; Nuclear PR staining (%), n=18; SBR grade, n=9; KI67 classes, n=209; Mitotic index class, n=42; Ductal carcinoma in situ, n=43; % stromal lymphocytes, n=57; % intra-tumoral lymphocytes, n=57; Lymphovascular invasion, n=256; NAC regimen, n=1; Lymphovascular invasion postNAC, n=142; RCB index, n=57; RCB classes, n=57; % stromal lymphocytes postNAC, n=57; % intra-tumoral lymphocytes postNAC, n=197.
Abbreviations: BMI= body mass index; T= tumor; N= node; ER= estrogen receptor; PR= progesterone receptor; SBR= grade Scarff-Bloom and Richardson; BC= Breast cancer; NAC=neoadjuvant chemotherapy; anthra-taxans= anthracyclines and taxanes; anthra= anthracyclines; RCB= Residual Cancer Burden.
The "n" denotes the number of patients. In case of categorical variables, percentages are expressed between brackets. In case of continuous variables, mean value is reported. In case of nonnormal continuous variables, median value is reported, with interquartile range between brackets.

Table S2: Association of clinical and pathological pre and post-NAC parameters with PD-L1-TC expressio after univariate and multivariate analysis in the whole population.
Abbreviations: BMI=body mass index; ER=oestrogen receptor; PR=progesterone receptor; TNBC= triple negative breast cancer ; str TILs= stromal tumor-infiltrating lymphocytes ; IT TILs= intratumoral-infiltrating lymphocytes ; NAC=neoadjuvant chemotherapy ; AC=anthracyclines ; pCR=Pathologic complete response.

Table S3: Studies on PD-L1 expression in BC RD
Abbreviations: breast cancer (BC), triple-negative breast cancer (TNBC), hazard ratio (HR), confidence interval (CI), not available (NA), relative risk (RR)

Table S1: Patient characteristics among the whole population and by pCR status.

Characteristics	Class	No pCR	pCR	*p*
n=		236	139	
**Baseline characteristics**				
**Age**		48.75 (10.41)	47.67 (10.05)	0.327
**Family history**	No	188 (79.7)	94 (68.1)	0.017
	Yes	48 (20.3)	44 (31.9)	
**BRCA screening**	No	175 (74.2)	90 (64.7)	0.070
	Yes	61 (25.8)	49 (35.3)	
**Menopausal status**	Premenopausal	140 (59.6)	89 (65.4)	0.313
	Postmenopausal	95 (40.4)	47 (34.6)	
**BMI classes**	18.5-24.9	124 (52.5)	86 (62.3)	0.081
	<18.5	11 (4.7)	1 (0.7)	
	25-29.9	70 (29.7)	33 (23.9)	
	>=30	31 (13.1)	18 (13.0)	
**Smoking status**	Never	128 (69.6)	78 (64.5)	0.280
	Current	32 (17.4)	19 (15.7)	
	Former	24 (13.0)	24 (19.8)	
**Comorbidity**	No	114 (55.9)	72 (62.1)	0.337
	Yes	90 (44.1)	44 (37.9)	
**Clinicar T stage**	T0-T1	11 (4.7)	20 (14.4)	**0.003**
	T2	155 (65.7)	87 (62.6)	
	T3-T4	70 (29.7)	32 (23.0)	
**Clinical N stage**	N0	110 (46.6)	61 (43.9)	0.686
	N1-N2-N3	126 (53.4)	78 (56.1)	
**SBR grade**	Grade I-II	44 (19.2)	9 (6.6)	**0.002**
	Grade III	185 (80.8)	128 (93.4)	
**Mitotic index class**	[0,20)	82 (40.2)	33 (25.6)	**0.009**
	>=20	122 (59.8)	96 (74.4)	
**Ductal carcinoma in situ**	No	170 (82.5)	112 (88.2)	0.216
	Yes	36 (17.5)	15 (11.8)	
**% stromal lymphocytes**		20.00 [5.00, 30.00]	30.00 [15.00, 60.00]	**<0.001**
**% intra-tumoral lymphocytes**		5.00 [1.00, 5.00]	5.00 [5.00, 20.00]	**<0.001**
**Lymphovascular invasion**	No	61 (71.8)	21 (60.0)	0.297
	Yes	24 (28.2)	14 (40.0)	
**NAC regimen**	anthra-taxans	173 (73.3)	114 (82.0)	0.227
	anthra	46 (19.5)	17 (12.2)	
	taxanes	1 (0.4)	0 (0.0)	
	others	16 (6.8)	8 (5.8)	
**Post-NAC characteristics**				
**N stage**	0	141 (59.7)	139 (100.0)	**<0.001**
	[1-3]	54 (22.9)	0 (0.0)	
	[4-9]	33 (14.0)	0 (0.0)	
	10 and more	8 (3.4)	0 (0.0)	
**RCB index**		2.39 (0.93)	0.00 (0.00)	**<0.001**
**RCB classes**	RCB-0	0 (0.0)	123 (100.0)	**<0.001**
	RCB-I	23 (11.7)	0 (0.0)	
	RCB-II	131 (66.8)	0 (0.0)	
	RCB-III	42 (21.4)	0 (0.0)	
**Lymphovascular invasion pos**	No	125 (66.1)	45 (100.0)	**<0.001**
	Yes	64 (33.9)	0 (0.0)	
**% stromal lymphocytes post-NAC**		15.00 [5.00, 30.00]	5.00 [5.00, 10.00]	**<0.001**

Missing data: Family history, n=1; Menopausal status, n=4; BMI classes, n=1; Smoking status, n=71; Comorbidity, n=56; Nuclear ER staining (%), n=20; Nuclear PR staining (%), n=18; SBR grade, n=9; KI67 classes, n=209; Mitotic index class, n=42; Ductal carcinoma in situ, n=43; % stromal lymphocytes, n=57; % intra-tumoral lymphocytes, n=57; Lymphovascular invasion, n=256; NAC regimen, n=1; Lymphovascular invasion postNAC, n=142; RCB index, n=57; RCB classes, n=57; % stromal lymphocytes postNAC, n=57; % intra-tumoral lymphocytes postNAC, n=197.
Abbreviations: BMI= body mass index; T= tumor; N= node; SBR= grade Scarff-Bloom and Richardson; BC= Breast cancer; NAC=neoadjuvant chemotherapy; anthra-taxans= anthracyclines and taxanes; anthra= anthracyclines; RCB= Residual Cancer Burden.
The "n" denotes the number of patients. In case of categorical variables, percentages are expressed between brackets. In case of continuous variables, mean value is reported. In case of nonnormal continuous variables, median value is reported, with interquartile range between brackets.

Table S2: Association of clinical and pathological pre and post-NAC parameters with PD-L1-TC expressio after univariate and multivariate analysis in the whole population

					Univariate				Multivariate		
**Variable**	**Class**	**Nb total**	**Nb in model**	**Events**	**HR**	**CI**	**RCH**	***p***	**HR**	**CI**	***p***
**Pre-NAC parameters**											
**Age**				31	1	[0.96 - 1.04]		0.959			
**Family history**	No	68	68	24	1		35.3 %				
	Yes	21	21	7	0.92	[0.31 - 2.53]	33.3 %	0.869			
**BRCA screening**	No	56	56	20	1		35.7 %				
	Yes	33	33	11	0.9	[0.36 - 2.21]	33.3 %	0.82			
**Menopausal status**	Premenopausal	51	51	18	1		35.3 %				
	Postmenopausal	38	38	13	0.95	[0.39 - 2.3]	34.2 %	0.915			
**BMI classes**	18.5-24.9	47	NA	13			27.7 %				
	<18.5	2	NA	0			0%				
	25-29.9	22	NA	10			45.5 %				
	>=30	18	NA	8			44.4 %				
**Smoking status**	Never	61	61	19	1		31.1 %				
	Current	12	12	4	1.11	[0.27 - 3.98]	33.3 %	0.882			
	Former	12	12	6	2.21	[0.62 - 7.96]	50%	0.215			
**Comorbidity**	No	35	35	10	1		28.6 %				
	Yes	42	42	16	1.54	[0.59 - 4.12]	38.1 %	0.38			
**Clinicar T stage**	T0-T1	6	6	4	1		66.7 %				
	T2	62	62	23	0.29	[0.04 - 1.63]	37.1 %	0.177	0.41	[0.05 - 2.68]	0.36
	T3-T4	21	21	4	0.12	[0.01 - 0.81]	19%	**0.038**	0.16	[0.01 - 1.43]	0.114
**Clinical N stage**	N0	38	38	13	1		34.2 %				
	N1-N2-N3	51	51	18	1.05	[0.43 - 2.57]	35.3 %	0.915			
**SBR grade**	Grade I-II	12	12	1	1		8.3 %				
	Grade III	75	75	29	6.93	[1.25 - 130.09]	38.7 %	0.07	3.87	[0.55 - 79.01]	0.24
**Mitotic index**				31	1.01	[0.99 - 1.03]		0.185			
**Mitotic index status**	[0,20)	34	34	10	1		29.4 %				
	>=20	48	48	18	1.44	[0.57 - 3.78]	37.5 %	0.448			
**Ductal carcinoma in situ**	No	83	83	30	1		36.1 %				

	Yes	6	6	1	0.35	[0.02 - 2.33]	16.7 %	0.353			
**% stromal lymphocytes**				31	1.02	[1 - 1.04]		0.071	1	[0.96 - 1.05]	0.812
**% intra-tumoral lymphocytes**				31	1.04	[1 - 1.09]		0.086	1.02	[0.95 - 1.1]	0.599
**Lymphovascular invasion**	No	2	NA	1			50%				
	Yes	1	NA	1			100%				
**BC surgery**	Lumpectomy	59	59	18	1		30.5 %				
	Mastectomy	30	30	13	1.74	[0.7 - 4.35]	43.3 %	0.232			
**NAC regimen**	anthra-taxans	85	NA	30			35.3 %				
	anthra	3	NA	1			33.3 %				
	taxanes	1	NA	0			0%				
**Chemotherapy setting**	NAC	59	59	19	1		32.2 %				
	NAC and adjuvant	30	30	12	1.4	[0.56 - 3.49]	40%	0.466			
**Post-NAC parameters**											
**N stage post-NAC**	0	53	NA	18			34%				
	[1-3]	22	NA	5			22.7 %				
	[4-9]	13	NA	8			61.5 %				
	10 and more	1	NA	0			0%				
**RCB classes**	RCB-I	15	15	3	1		20%				
	RCB-II	53	53	16	1.73	[0.47 - 8.34]	30.2 %	0.441	1.02	[0.21 - 5.91]	0.978
	RCB-III	19	19	11	5.5	[1.26 - 30.51]	57.9 %	**0.032**	2.94	[0.48 - 21.7]	0.259
**KI67 status post-NAC**	[0,20)	51	51	17	1		33.3 %				
	>=20	38	38	14	1.17	[0.48 - 2.82]	36.8 %	0.731			
**Mitotic index post-NAC (%)**				31	1.01	[1 - 1.03]		**0.035**	1.01	[0.99 - 1.02]	0.431
**Mitotic index status post-NAC**	[0,20)	43	43	14	1		32.6 %				
	>=20	35	35	12	1.08	[0.42 - 2.79]	34.3 %	0.872			
**% stromal lymphocytes post-NAC**				31	1	[0.98 - 1.03]		0.758			
**% intra-tumoral lymphocytes post-NAC**				31	0.99	[0.91 - 1.06]		0.718			
**Lymphovascular invasion post-NAC**	No	54	54	22	1		40.7 %				
	Yes	23	23	6	0.51	[0.16 - 1.45]	26.1 %	0.225			

Abbreviations: BMI=body mass index; TNBC= triple negative breast cancer ; str TILs= stromal tumor-infiltrating lymphocytes ; IT TILs= intratumoral-infiltrating lymphocytes ; NAC=neoadjuvant chemotherapy ; AC=anthracyclines ; pCR=Pathologic complete response.

First author	Journal	Year	Country	n clinical studies	n patients	PDL-1 prevalence	Association with clinicopathological characteristics	PDL1 and pCR	PDL1 and DFS	PDL1 and OS
Wang	The Breast Journal	2017	China	9	8583	25.8% (2141/8288)	Ductal BC (p=0.037)<br>High tumor grade (p=0.0001)<br>ER negative (p=0.0001)<br>PR negative (p=0.0001)<br>Her2+ (p=0.001)<br>Basal like (p=0.0001)	-	HR = 1.122<br>95% CI [0.878; 1.434]<br>p = 0.357	HR 1.573<br>95% CI [1.01; 2.45]<br>p = 0.045
Kim	BMC Cancer	2017	Korea	6	7877<br>(827 TNBC)	-	Tumor and immune cells:<br>Lymph node metastasis (p=0.06)<br>High tumor grade (p<0.001)	-	HR = 1.36<br>95% CI [1.03; 1.79]<br>p = 0.03	HR = 1.908<br>95% CI [0.91; 4.00]<br>p = 0.09
Zhang	Oncotarget	2017	China	5	2546	21.7% to 56.6%	Lymph node metastasis (p=0.02)<br>High tumor grade (p<0.001)<br>ER negative (p=0.008)<br>TNBC (p<0.001)	-	-	HR=1.76<br>95% CI [1.09; 2.82]<br>p=0.02
Huang	Breast Cancer Research and Treatment	2019	China	41	14367	-	Ductal BC (p=0.03)<br>Large tumor size (p=0.05)<br>High tumor grade (p<0.00001)<br>ER negative (p=0.0009)<br>PR negative (p=004)<br>High Ki67 (p=0.01)<br>TNBC (p=0.0001)<br>High TILS (p<0.00001)	Tumor c. : RR = 1.64<br>95% CI [0.99–2.73]<br>p = 0.05<br>(n=1085)	Tumor c. :<br>HR = 1.43<br>95% CI [1.21; 1.70]<br>p < 0.0001 (n=4349)<br>Immune c. :<br>HR = 0.45<br>95% CI [0.28; 0.73]<br>p = 0.001	Tumor c. : HR = 1.58<br>95% CI [1.14; 2.20]<br>p = 0.006 (n=5890)<br>Immune c. :<br>HR = 0.41<br>95% CI [0.27; 0.63]<br>p < 0.0001
Li	Medicine	2019	China	19	12505	6.4% to 76.4%	Lymph node metastasis (p<0.001)<br>High tumor grade (p<0.001)<br>Negative HR (p<0.001)<br>High Ki67 (p<0.001)<br>High TILS (p<0.001)	-	HR = 1.31<br>CI95% [1.14; 1.51]<br>p = 0.0002	HR = 1.52<br>CI95% [1.14;2.03]<br>p=0.004
Matikas	Clinical Cancer Research	2019	Sweden	38	9500	In the TNBC subgroup:<br>tumor cells 41% (n =2740)<br>immune c. 48% (n=1313)<br>tumor and immune c. 46% (n=903)	-	-	Tumor c. overall :<br>HR 1.62<br>95% CI [1.14–2.33]<br>p=0.008<br>Immune c. TNBC :<br>HR =0.61<br>95% CI [0.51–0.73]<br>p < 0.001 (n=969)	Tumor c. overall :<br>HR = 1.93<br>95% CI [1.20; 3.09]<br>p=0.006<br>Immune c. TNBC :<br>HR = 0.53<br>95% CI [0.39; 0.73]<br>p < 0.001
Lotfinejad	Diagnostics	2020	Iran	7	1152	-	No associations	-	HR = 0.65<br>95% CI [0.37; 1.16]<br>p = 0.15	HR = 0.56<br>95% CI [0.32 to 1.01]<br>p = 0.056

Table S3. Recent meta-analyses of the prognostic value of PD-L1 (IHC) in BC *Abbreviations:* breast cancer (BC), estrogen receptor (ER), progesterone receptor (PR), hormone receptor (HR), triple-negative breast cancer (TNBC), tumor-infiltrating lymphocytes (TILS), cells (c.), hazard ratio (HR), confidence Interval (CI), relative risk (RR), pathological complete response (pCR), overall survival (OS, disease-free survival (DFS)

Supplementary Figures

Fig. S1 Immunohistochemical analysis of PD-L1 in surgical specimens (Cell Signaling #13684S (E1L3N®) XP® rabbit mAb) staining. **A,** Positive control, breast carcinoma cells cytoblock, PD-L1 strong membrane expression, reported at ≥50% (x400); **B,** Positive control, Tonsil, PD-L1 strong expression of epithelial and microenvironment cells, reported at ≥50% (x200); **C,** residual invasive breast cancer obtained after NAC, strong membrane and cytoplasmic PDL1 expression by tumor and microenvironment cells, reported at ≥50% (x200); **D,** residual invasive breast cancer obtained after NAC, strong PDL1 expression by immune cells of the tumor microenvironment, reported at ≥50% (x400); **E,** residual invasive breast cancer obtained after NAC, strong PDL1 by breast carcinoma cells, reported at ≥50% (x400).

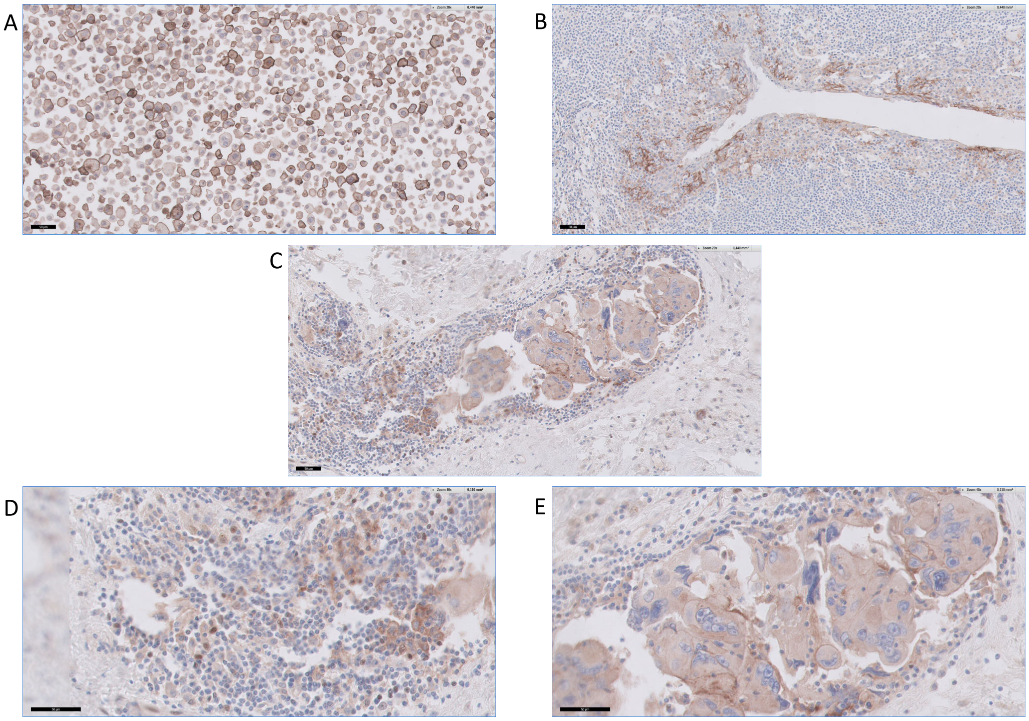

Fig. S2: Pre-NAC and post-NAC stromal immune infiltration rates in the whole population and by BRCA status. Waterfall plot representing the variation of TIL levels according to PD-L1-TC and PD-L1-IC; each bar represents one sample, and samples are ranked by increasing order of TIL level change. Paired samples for which no change was observed have been removed from the graph. **A,** by PD-L1_TC. B, by PD-L1-IC.

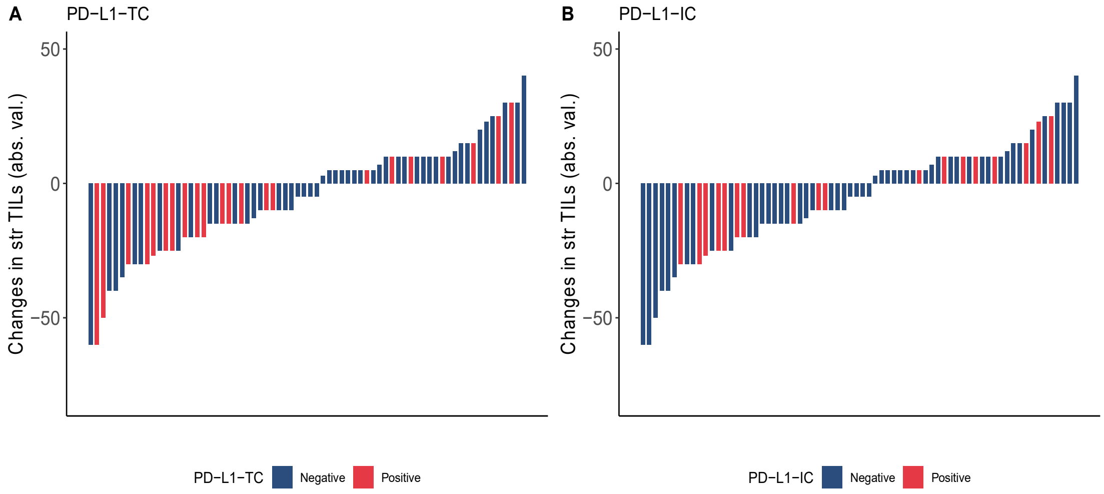
A
PD−L1−TC
50
0
−50
Changes in str TILs (abs. val.)
PD−L1−TC
Negative
Positive
B
PD−L1−IC
50
0
−50
Changes in str TILs (abs. val.)
PD−L1−IC
Negative
Positive
